# Effects of Soil Properties on the Demography of Bud Banks in Different Degraded Meadows on the Qinghai–Tibet Plateau

**DOI:** 10.3390/plants15101462

**Published:** 2026-05-11

**Authors:** Yuan Li, Qian Zhao, Shuihong Chen, Gensheng Bao

**Affiliations:** 1 Academy of Animal Science and Veterinary, Qinghai University, Xining 810003, China; liy19991226@163.com (Y.L.); ys240909000837@qhu.edu.cn (Q.Z.); 2 Key Laboratory of Qinghai-Tibetan Plateau Superior Forage Germplasm Research, Qinghai Academy of Animal and Veterinary Medicine, Xining 810016, China; 3 Laboratory for Protection and Genetic Improvement of Qinghai Tibet Plateau Germplasm Resources (Co-Construction by Ministry and Province), Academy of Agriculture and Forestry Sciences, Qinghai University, Xining 810003, China; cshdky@126.com; 4 College of Life Science and Technology, Tarim University, Alar 843300, China

**Keywords:** degraded alpine meadow, overgrazing, bud bank, soil physicochemical properties, soil microbial biomass

## Abstract

Although bud banks are key components of vegetation regeneration in degraded alpine meadows, their relationships with soil conditions on the Qinghai–Tibet Plateau remain insufficiently understood. In this study, we investigated bud bank composition and density, plant functional group biomass, soil physicochemical properties, and soil microbial biomass across five degradation stages of alpine meadows in a long-term controlled grazing experiment. Field sampling was conducted in mid-August 2021, and the relationships between bud bank densities, plant biomass, and soil variables were evaluated using comparative statistical analyses, redundancy analysis, and structural equation modeling. Bud bank density increased from non-degraded to moderately degraded meadows, reaching 3075 buds m^−2^, but declined sharply in severely degraded meadows to 183 buds m^−2^. Regarding distinct bud types, rhizome and tiller bud densities peaked in moderately degraded alpine meadows (1217 and 1750 buds m^−2^, respectively), whereas dicot bud density peaked in lightly degraded meadows. Bud bank density was positively associated with higher soil moisture content and negatively associated with increased soil bulk density. Moreover, bud bank density was positively correlated with soil organic carbon, total phosphorus, ammonium nitrogen, and soil microbial biomass carbon, nitrogen, and phosphorus. Our findings indicate that soil conditions may favor the maintenance of high bud bank density in moderately degraded meadows with high soil moisture, low bulk density, and more nutrient-rich soil conditions in moderately degraded meadows. Overall, our results indicate that alpine meadow degradation influences belowground regenerative capacity through changes in soil conditions and associated shifts in bud bank dynamics. Therefore, assessments and restoration of degraded alpine meadows should consider bud bank persistence in addition to aboveground vegetation characteristics.

## 1. Introduction

A bud bank is a population of buds capable of vegetative propagation, which primarily consists of rhizome, tiller, and dicot buds [1,2]. Bud banks are regarded as key vegetative propagators that facilitate the recovery and regeneration of aboveground plant populations following abiotic and biotic disturbances in perennial grassland ecosystems [3,4,5], and can serve as integrative indicators of plant population responses to disturbance intensity and adaptations to environmental pressures [6,7]. The composition and density of belowground bud banks can function as practical proxy indicators for predicting successional trajectories in natural vegetation [8]. Grassland ecosystems, particularly those dominated by perennial plant species, rely primarily on the regenerative capacity of belowground bud banks rather than on sexual reproduction to maintain plant community structure, population recruitment, and long-term community dynamics [9,10]. For example, Benson and Hartnett [11] demonstrated that over 99% of established stems originated from vegetative propagation via rhizomes and tillers, in contrast to seedling recruitment, which constituted less than 1% of all established stems. Together, these studies indicate that bud banks are central to the recovery of perennial grasslands after abiotic and biotic disturbance [12], including seasonal fire [13,14], drought [15], and grazing [5,13].

Empirical evidence indicates that the different degradation stages of perennial grassland partly reflect the disturbance intensity of herbivore activities, with the disturbance and trampling frequency of herbivores profoundly impacting the demography of bud banks [8,13,16]. In the study by Yang et al. [16], rhizome buds dominated the bud bank composition in alpine meadows that were not deteriorated, whereas tiller buds predominated in meadows that were only mildly damaged. Furthermore, the rhizome density of sedges decreased with enhanced degradation, while the corm density of forbs increased with intensification of the degradation level. Similarly, VanderWeide and Hartnett [8] indicated that the rhizome density of sedges markedly decreased under the combined effects of grazing and water deficiency. In tallgrass prairies, bud banks can be depleted by herbivore grazing—an effect attributable to consumption and physical damage, as reported by Dalgleish and Hartnett [13]. These studies consistently support strong grazing effects on bud bank composition, but they mainly emphasize changes in bud bank size or dominant bud types. Much less attention has been paid to how degradation-related shifts in soil conditions, plant functional groups, and species turnover jointly shape bud bank dynamics in alpine meadows. Grazing also alters soil physical traits, nutrient availability, and soil microbial biomass, and increased trampling can increase bulk density while reducing nutrient availability in soil [17]; however, few studies have explicitly linked these soil changes to bud bank composition and density across controlled degradation gradients.

Soil physicochemical properties not only largely determine the species composition and vegetation dynamics in natural ecosystems, but are also regarded as a decisive factor in the type and density of bud banks [5,18,19]. For example, Wu et al. [19] indicated that soil water content is a crucial limiting factor in determining the density of rhizomes and root-sprouting buds in the interdune lowlands of active dunes, while rhizome and root-sprouting buds in the interdune lowlands of fixed dunes are largely dependent on the soil total carbon. Similarly, a significant positive correlation was observed by Ding et al. [20] between soil moisture content and both the total belowground bud bank density and short-rhizome bud density in the alpine meadows of the Zoige Plateau. Furthermore, Wu et al. [21] demonstrated that nitrogen addition markedly increased the total density of the bud bank, while soil water deficiency was regarded as the key factor restricting its total density, especially for tiller and corm buds from grasses and forbs, respectively. Furthermore, soil compaction significantly reduced the density of rhizome buds, and field capacity negatively affected the formation and regeneration of tuber buds for lignotuber species [22]. Soil available nutrients are a critical regulator of bud bank dynamics. Klimeš and Klimešová [23] reported that a root-sprouting species produced more buds and shoot biomass in nutrient-rich soil than in nutrient-poor soil. Ott et al. [5] concluded that elevated available nitrogen in soil enhanced active-bud density per tiller in C_3_ grasses. Sun et al. [24] observed that exogenous nitrogen increased clonal fragment biomass. These studies show that soil moisture, soil compaction, and nutrient availability can regulate bud bank formation and persistence, but they also reveal a tendency to consider soil drivers in isolation.

Recent studies have suggested that soil effects on plant communities should be interpreted together with plant functional traits and soil–plant feedback processes. Functional differences among plant groups can modify trait–productivity relationships along soil moisture gradients [25], and plant functional traits have been shown to predict soil microbial diversity in alpine meadows [26]. Moreover, plant–soil feedback responses to environmental stress may be strongly species-specific and cannot always be explained solely by root traits [27,28]. Although these studies do not directly examine belowground bud banks, they indicate that plant responses to edaphic variation are mediated by both trait differences and plant–soil interactions. Therefore, in degraded alpine meadows, variations in bud bank composition and density are likely to be influenced not only by soil physicochemical conditions, but also indirectly by shifts in plant functional composition and species-specific regeneration strategies.

Alpine meadows constitute a vital component of the grassland ecosystem in the Qinghai–Tibet Plateau (QTP), performing critical ecological services such as windbreak, sand fixation, soil and water conservation, and contributions to global climate regulation [29,30]. However, estimates from recent research reveal that the extent of degraded alpine meadows on the QTP ranges from 19% to 60%—a phenomenon significantly influenced by the interplay of climatic change and anthropogenic pressures [31,32,33]. The ongoing degradation of alpine meadows has triggered a series of adverse changes in grassland ecosystems, including reduced vegetation cover, diminished species diversity, deteriorated soil structure, and depleted soil nutrients [34,35,36]. Alpine meadow degradation is largely driven by overgrazing, characterized by livestock overstocking rates of 27% to 89% [37]. Grazing has not only decreased plant coverage and productivity but also deteriorated soil properties [38,39]. A range of effective strategies has been identified in the literature for the rehabilitation of degraded alpine meadows [40], with the most effective measures including fencing enclosures and artificial restoration through fertilization and reseeding [33]. Compared with the negligible effects of reseeding in restoring degraded grasslands, bud banks are recommended as fundamental drivers for regenerating the plant community when the grassland suffers from detrimental disturbance [5]. However, soil–bud bank interactions are insufficiently understood in degraded alpine meadows, particularly under long-term controlled grazing. Compared with previous research, the present study advances this topic by explicitly linking the degradation stage, soil physicochemical properties, soil microbial biomass, bud bank composition, and plant functional group biomass within a single controlled grazing framework.

Our study aims to investigate how soil physicochemical properties regulate the dynamics of different bud bank components across successive degradation stages of alpine meadows. We hypothesized that (1) grazing-induced meadow degradation regulates belowground bud bank density and composition along the degradation gradient, with low-intensity herbivore disturbance promoting bud bank accumulation and severe degradation suppressing bud renewal; (2) soil properties mediate these changes, with higher nutrient availability and microbial biomass promoting bud formation and persistence, whereas adverse soil physical conditions constrain bud development; (3) these effects differ between grasses, sedges, and forbs, as plant functional groups vary in their clonal growth strategies and sensitivity to soil conditions; and (4) changes in bud bank density and composition are also partly driven by shifts in plant species composition, reflecting interspecific differences in bud production capacity and clonal regeneration strategies. To verify these hypotheses, we performed a field investigation to record the type and density of bud banks and examine soil properties at different meadow degradation stages. Our study focuses on two key scientific questions: (1) how do the belowground bud bank type and density change at different degradation stages of alpine meadows? (2) How do specific soil variables (soil moisture, bulk density, and nutrient availability) quantitatively influence bud bank type and density along the degradation gradient? Clarifying these relationships is essential for understanding the mechanistic links between soil properties and bud bank dynamics, thus informing effective restoration strategies for degraded alpine meadows on the Qinghai–Tibet Plateau by enhancing bud bank regeneration capacity.

## 2. Results

### 2.1. Biomass of Plant Functional Groups in Different Degraded Alpine Meadows

**Figure 1:**
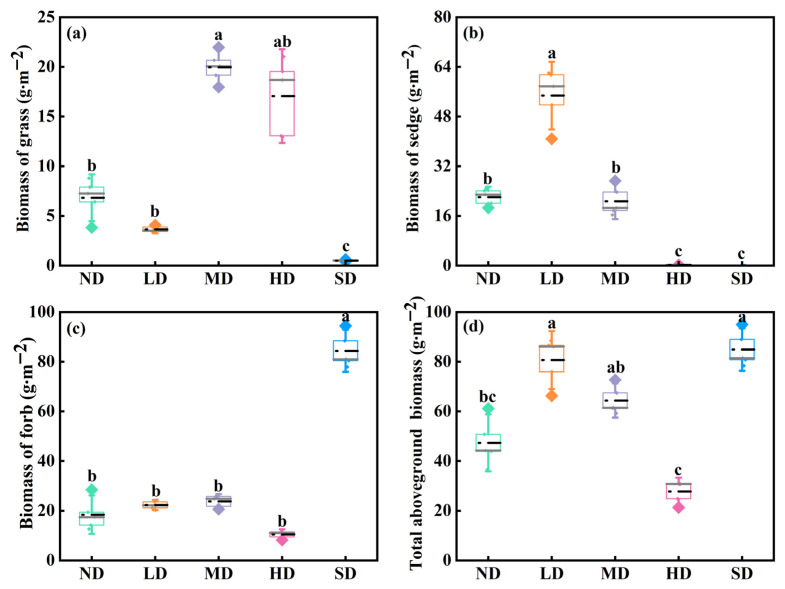
Aboveground biomass of grasses (**a**), sedges (**b**), forbs (**c**), and total vegetation (**d**) across five degradation stages of alpine meadows. Different lowercase letters indicate that the aboveground biomass of identical functional groups was significantly different at the 0.05 level among the different degradation stages.

Aboveground biomass differed significantly among the degraded alpine meadows for all plant functional groups and for total vegetation biomass (Figure 1; *p* < 0.05). The grass biomass of MD meadows was significantly higher than that of ND, LD, and SD meadows (Figure 1a; *p* < 0.05). Sedge biomass was highest in LD meadows and significantly greater than that in the other degradation stages; in contrast, sedge biomass was lowest in HD and SD meadows (Figure 1b; *p* < 0.05). Conversely, the forb biomass of SD meadows was the maximum among the degraded meadows (Figure 1c; *p* < 0.05). Total aboveground biomass was highest in LD and SD meadows, intermediate in MD meadows, and lowest in ND and HD meadows (Figure 1d; *p* < 0.05).

### 2.2. Density of Bud Banks in Different Degraded Alpine Meadows

**Figure 2:**
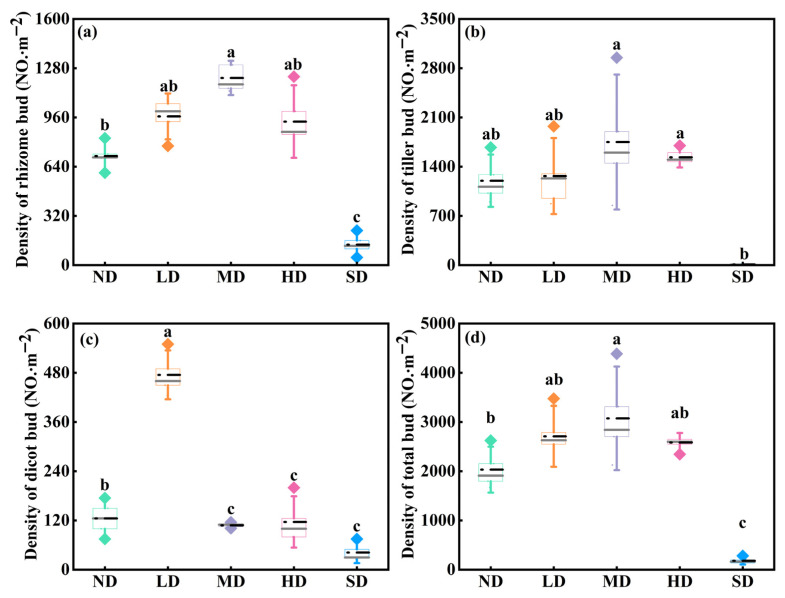
Types and densities of bud banks in degraded alpine meadows. Density of rhizome buds (**a**), tiller buds (**b**), dicot buds (**c**), and total buds (**d**) across five degradation stages of alpine meadows. Different lowercase letters indicate that the density of identical bud types was significantly different at the 0.05 level among the different degradation stages.

Belowground bud bank density varied significantly among various degradation stages (Figure 2; *p* < 0.05). The highest densities of rhizome, tiller, and total buds were all significantly higher in MD meadows than in SD meadows, reaching 1217, 1750, and 3075 buds m^−2^ in MD meadows, respectively, compared with 133, 8, and 183 buds m^−2^ in SD meadows (Figure 2a,b,d; *p* < 0.05). In contrast, the density of dicot buds was highest in LD meadows, whereas no significant difference was detected between MD, HD, and SD meadows (Figure 2c; *p* > 0.05).

### 2.3. Soil Physical Properties and Nutrient Levels in Different Degraded Alpine Meadows

**Figure 3:**
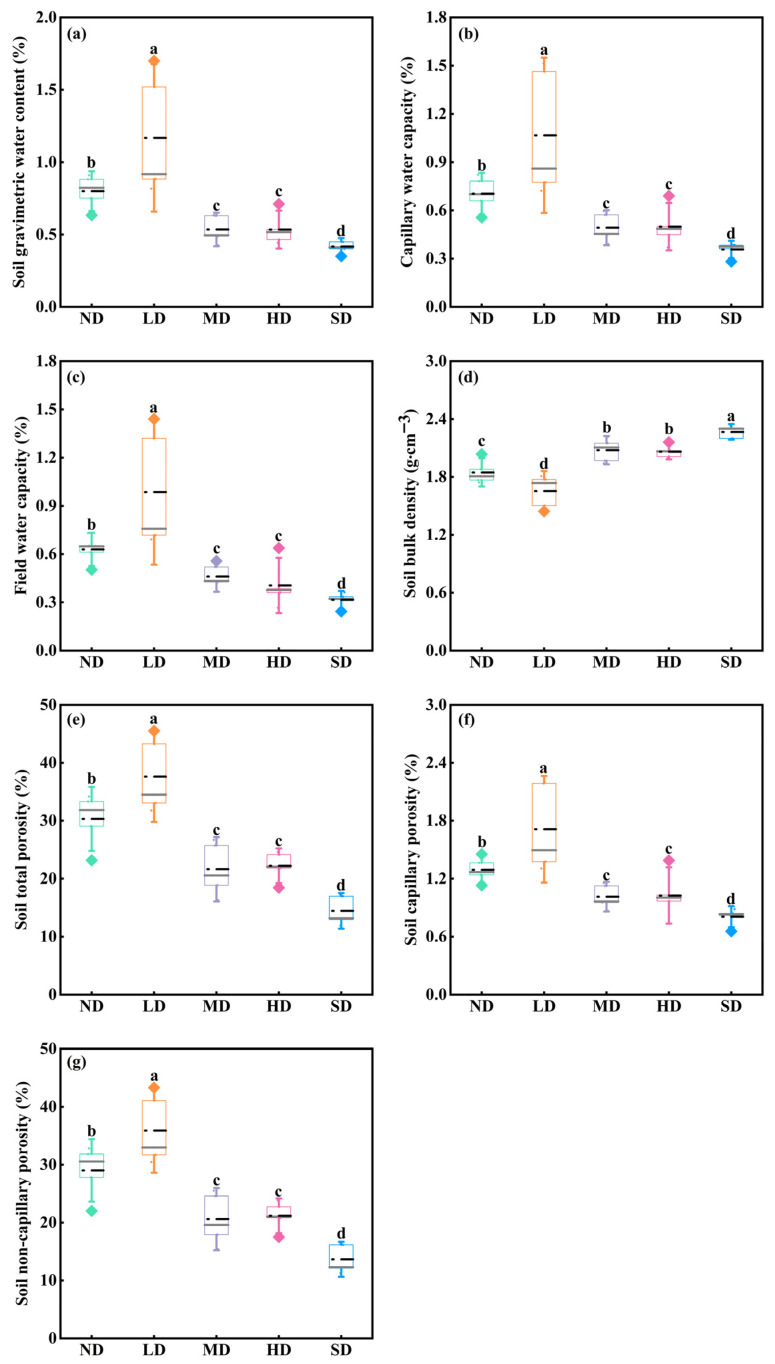
Changes in soil physical properties in different degraded alpine meadows, including soil gravimetric water content (SWC, (**a**)), capillary water capacity (CWC, (**b**)), field water capacity (FWC, (**c**)), soil bulk density (BD, (**d**)), soil total porosity (STP, (**e**)), soil capillary porosity (CP, (**f**)), and soil non-capillary porosity (NCP, (**g**)). Different lowercase letters indicate significant differences among degradation stages for the same variable at the 0.05 level.

Soil physical properties varied significantly among degradation stages (Figure 3; *p* < 0.05). In general, soil water content and porosity variables were highest in LD meadows and lowest in SD meadows, whereas soil bulk density showed the opposite trend. Specifically, SWC, CWC, FWC, STP, CP, and NCP were all significantly higher in LD meadows than in MD, HD, and SD meadows (Figure 3a–c,e–g; *p* < 0.05). In contrast, BD was highest in SD meadows but lowest in LD meadows (Figure 3d).

**Figure 4:**
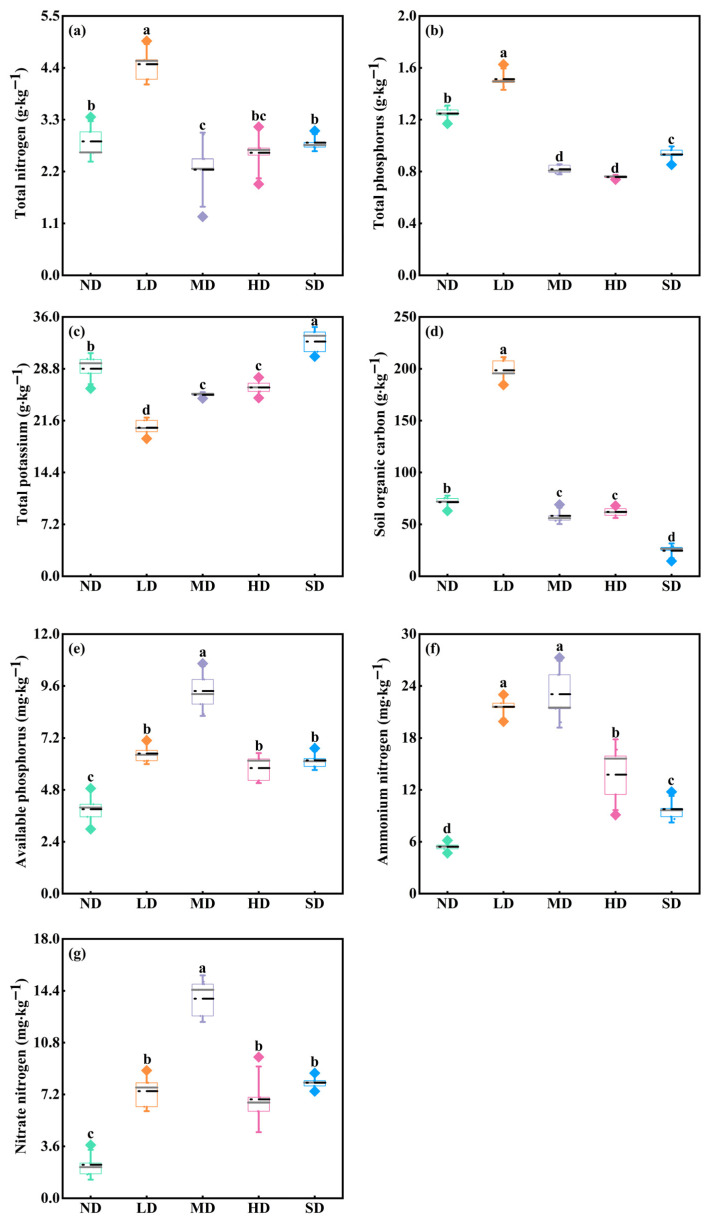
Changes in soil chemical traits in different degraded stages of alpine meadows, including total nitrogen (TN, (**a**)), total phosphorus (TP, (**b**)), total potassium (TK, (**c**)), soil organic carbon (SOC, (**d**)), available phosphorus (AP, (**e**)), ammonium nitrogen (NH_4_^+^-N, (**f**)), and nitrate nitrogen (NO_3_^−^-N, (**g**)). Different lowercase letters indicate significant differences among degradation stages for the same variable at the 0.05 level.

Soil chemical properties differed significantly among degradation stages (Figure 4; *p* < 0.05). TN and TP were highest in LD meadows, whereas TK was highest in SD meadows (Figure 4a–c). SOC also reached its maximum in LD meadows, where it was 177.87%, 241.02%, 220.23%, and 709.25% higher than in ND, MD, HD, and SD meadows, respectively (Figure 4d; *p* < 0.05). Regarding the available soil nutrients, AP, NH_4_^+^-N, and NO_3_^−^-N were highest in MD meadows and lowest in ND meadows (Figure 4e–g; *p* < 0.05). No significant differences in AP and NO_3_^−^-N were detected between LD, HD, and SD meadows (Figure 4e,g; *p* > 0.05).

### 2.4. Soil Microbial Biomass in Different Degraded Alpine Meadows

**Figure 5:**
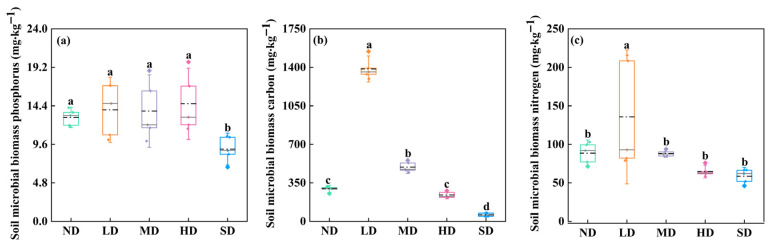
Changes in soil microbial biomass in different degraded alpine meadows, including soil microbial biomass phosphorus (SMBP, (**a**)), soil microbial biomass carbon (SMBC, (**b**)), and soil microbial biomass nitrogen (SMBN, (**c**)). Different lowercase letters indicate significant differences among degradation stages for the same variable at the 0.05 level.

Soil microbial biomass differed significantly among degradation stages (Figure 5; *p* < 0.05). SMBP in SD meadows was significantly lower than that in ND, LD, MD, and HD meadows (Figure 5a; *p* < 0.05), and no significant differences were detected between ND, LD, MD, and HD meadows (Figure 5a; *p* > 0.05). SMBC reached its maximum in LD meadows (1385.56 mg kg^−1^) compared with those in ND (295.98 mg kg^−1^), MD (493.88 mg kg^−1^), HD (240.53 mg kg^−1^), and SD (60.04 mg kg^−1^) (Figure 5b; *p* < 0.05). Similarly, SMBN was significantly higher in LD meadows than in ND, MD, HD, and SD meadows (Figure 5c, *p* < 0.05), whereas no significant differences were observed between the other four degraded alpine meadows (Figure 5c, *p* > 0.05).

### 2.5. Correlations Among Plant Functional Groups Biomass, Bud Density, Soil Physicochemical Properties, and Microbial Biomass

**Figure 6:**
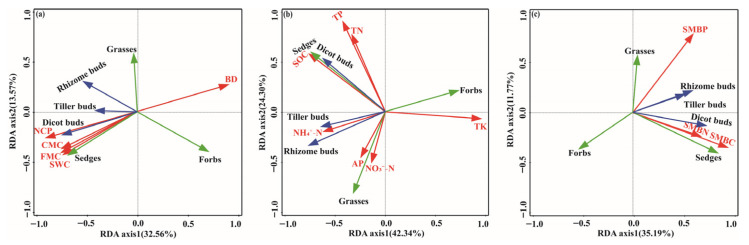
Redundancy analysis (RDA) of plant functional groups, bud banks, soil physical and chemical properties, and microbial biomass. RDA of plant functional groups, bud banks, and soil physical properties (**a**), soil chemical properties (**b**), and soil microbial biomass (**c**).

Redundancy analysis showed clear associations between bud bank traits, plant functional groups, and soil properties (Figure 6). Bud density and sedge biomass were positively correlated with soil physical properties, including NCP, CMC, FMC, and SWC, and negatively correlated with BD and forb biomass (Figure 6a). In contrast, forb biomass was negatively correlated with bud density, sedge, grass biomass, and soil physical properties but positively correlated with BD (Figure 6a). Similarly, rhizome bud density, tiller bud density, and grass biomass were positively correlated with AP, NH_4_^+^-N, and NO_3_^2212^-N. Dicot bud density and sedge biomass showed positive correlations with TP, TN, and SOC, while forb biomass was positively correlated with TK (Figure 6b). Furthermore, bud density and sedge biomass exhibited positive correlations with soil microbial biomass, including SMBP, SMBN, and SMBC, while forb biomass was negatively correlated with soil microbial biomass, grasses, and sedge biomass (Figure 6c).

**Figure 7:**
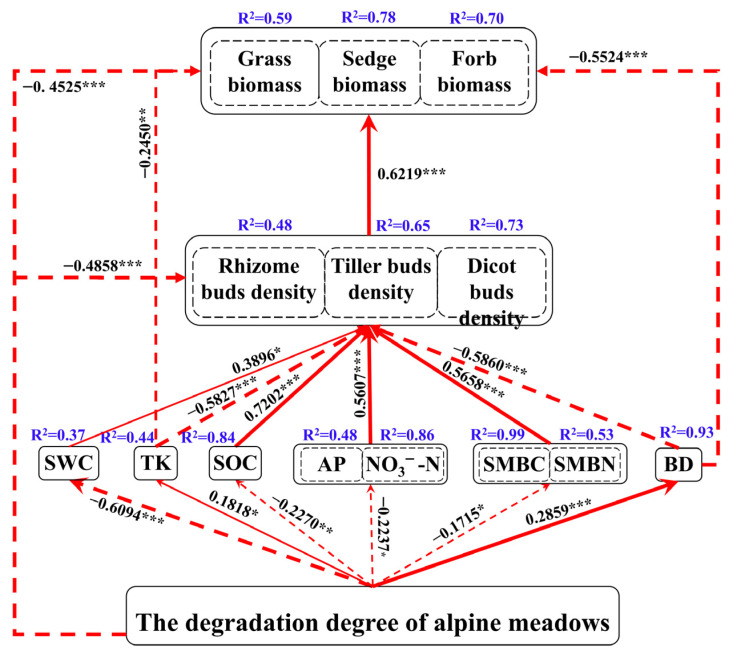
Structural equation model showing the relationships among plant functional group biomass, bud bank density, soil physicochemical properties, and soil microbial biomass across different degradation stages of alpine meadows. Red dotted line arrows indicate significant negative paths, and red solid arrows indicate significant positive paths. Values beside the arrows are standardized path coefficients, and arrow width is proportional to the strength of the path. R^2^ indicates the proportion of explained variance for each endogenous variable. The overall goodness of fit of the model was 0.7003 (* *p* < 0.05, ** *p* < 0.01, *** *p* < 0.001).

The structural equation model shows that intensified alpine meadow degradation had significant negative effects on SWC, SOC, available nutrients, soil microbial biomass, bud bank density, and plant functional group biomass, with path coefficients of −0.6094, −0.2270, −0.2233, −0.1715, −0.4858, and −0.4525, respectively. In contrast, degradation had significant positive effects on TK and BD, with path coefficients of 0.1818 and 0.2859, respectively (Figure 7; *p* < 0.05). Furthermore, the positive effects of SWC, SOC, soil nutrients, and SMB on the density of bud banks were 0.3896, 0.7202, 0.5607, and 0.5658, respectively. The negative effects of TK and BD on bud bank density were −0.5827 and −0.5860, respectively. Additionally, the bud bank density significantly contributed to plant vegetation biomass (Figure 7, *p* < 0.01), whereas TK and BD had significant negative effects on plant functional group biomass, with path coefficients of −0.2450 and −0.5524, respectively (Figure 7, *p* < 0.05). The model explained a substantial proportion of the variance in the endogenous variables, with a goodness of fit of 0.7003.

## 3. Discussion

### 3.1. Contributions of Bud Bank Composition to Plant Functional Group Biomass Among Different Degraded Alpine Meadows

#### 3.1.1. Contribution of Bud Bank Accumulation to Plant Functional Group Biomass Under Moderate Degradation

Our results show that rhizome, tiller, and total bud densities peaked in moderately degraded meadows (Figure 2d), suggesting that moderate grazing disturbance may enhance the regenerative capacity of palatable species through rapid resprouting from belowground bud banks [18,41]. Such bud banks may buffer the detrimental effects of grazing on daughter ramets, thereby promoting vegetation recovery after grazing [18,42]. Different contributions of specific bud types to functional group biomass provide further evidence supporting this hypothesis. As the primary regenerative organs of grass plants, both tiller buds and rhizome buds increased progressively from non-degraded to moderately degraded meadows, and this increase in bud density was accompanied by relatively high aboveground biomass at the early stages of degradation (Figure 1 and Figure 2). Corroborating our observations, Qian et al. [18] documented a marked increase in the proportion of tiller buds within the total bud bank in the early phases of degradation relative to later periods. Dalgleish and Hartnett [13] also suggested that grazing had a positive effect on increasing the stem density of grass by accelerating the emergence rate of tiller buds, while persistent grazing reduced tiller bud density. This is likely because tiller buds serve both storage and regeneration functions, and their density directly affects the grass population’s renewal capacity; conversely, higher-density tillers formed by mother grass demonstrated a detrimental effect on the renewal potential of the offspring grass population through belowground bud banks [43]. Taken together, these results suggest that moderate grazing is an effective regime for maintaining the resource balance between aboveground organs and the underground bud bank [20].

#### 3.1.2. Possible Mechanisms Underlying High Aboveground Biomass but Low Bud Bank Density Under Severe Degradation

The pattern observed in severely degraded meadows indicates that relatively high aboveground biomass does not necessarily imply a large or persistent bud bank. Although there was no significant difference in total aboveground biomass between lightly degraded and severely degraded meadows (Figure 1d), total bud density in lightly degraded meadows was 14.75 times higher than that in severely degraded meadows (Figure 2d). This mismatch suggests that maintaining aboveground biomass in severely degraded meadows may depend less on persistent belowground bud reserves and more on changes in plant functional group composition [18].

Several possible mechanisms may explain this pattern. First, selective foraging by herbivores may reduce dominant palatable grasses and sedges, whereas unpalatable forbs, such as *Ligularia virgaurea*, may become increasingly dominant under severe degradation because of their bitter taste and diverse secondary metabolites [44,45]. Previous studies have further suggested that root exudates from *L. virgaurea*, including alkaloids and terpenoids, may alter soil nutrient availability and microbial community composition, thereby potentially facilitating its persistence and expansion [46]. In addition, unpalatable forbs with effective reproductive strategies involving both seed production and vegetative regeneration may gain a competitive advantage in severely degraded meadows [47]. Second, Tibetan sheep may consume resprouting buds located near the soil surface at the tiller nodes of bunchgrasses, which may increase bud mortality and further reduce bud bank persistence under severe grazing pressure [48]. Third, many weedy forbs may maintain their populations through mixed regeneration strategies, including continuous seed input and opportunistic vegetative recruitment, rather than through the accumulation of dense and long-lived belowground bud banks [5,47,49]. Given that bud bank size depends on bud natality, outgrowth, and mortality, relatively high standing biomass in severely degraded meadows may still coexist with very low persistent bud bank density [5]. Moreover, severe degradation is usually associated with dry, compact, and nutrient-poor soil conditions, which may suppress bud formation and survival and thereby further limit bud bank persistence [5]. Therefore, the relatively high biomass observed in severely degraded meadows should not be interpreted as evidence of strong regenerative capacity, but rather as a consequence of shifts in species composition and reproductive strategy. These explanations should be regarded as possible mechanisms inferred from previous studies rather than direct evidence obtained in the present study, and require further verification in future work.

### 3.2. Non-Negligible Contributions of Soil Physical Properties and Nutrient Levels to Persistence of Bud Banks in Successive Degraded Meadows

#### 3.2.1. Soil Physical Properties and Bud Bank Persistence

As the primary substrate for plant growth, soil exerts a fundamental influence on bud bank demography through its physical properties [23,50]. Previous studies have shown that bud bank density is positively associated with soil water availability and negatively associated with aridity across multiple grassland types [50,51]. Supporting this observation, Qian et al. [52] and Wang et al. [53] likewise revealed the negative response of belowground bud bank density to elevated aridity. Given that the frequency and amount of precipitation are primary determinants of soil moisture, soil water content exerts a critical influence on the composition and density of the bud bank [19,54]. Our results were partly consistent with this general pattern, as soil water content was positively associated with bud bank density in the RDA and SEM analyses. However, the highest soil water content occurred in lightly degraded meadows, whereas total bud density peaked in moderately degraded meadows. This mismatch suggests that soil moisture alone cannot fully explain bud bank variation along the degradation gradient. A possible explanation is that lightly degraded meadows retained higher vegetation cover, which may have reduced soil evaporation and increased rainfall interception but simultaneously intensified aboveground competition for light and nutrients [55]. Under such conditions, more resources may have been allocated to aboveground organs rather than to belowground bud proliferation. In contrast, moderate grazing in MD meadows may have sufficiently reduced the competitive dominance of grasses and sedges to weaken aboveground competition while still maintaining soil conditions favorable for bud renewal. Therefore, our results suggest that soil moisture is an important, but not exclusive, factor in regulating bud bank persistence [54].

Soil bulk density and porosity have also been recommended as potential soil parameters to affect belowground bud densities [56,57]. Previous studies have reported that grazing and trampling increase soil bulk density, reduce porosity, and intensify soil drying and mechanical constraints on bud survival [58,59,60]. Consistent with these studies, our RDA results show that tiller and rhizome bud densities were positively associated with soil porosity and negatively associated with soil bulk density. This pattern indicates that tiller and rhizome buds are more sensitive than dicot buds to compact and dry soil conditions. In this sense, the decline in total bud density under severe degradation appears to be closely associated with the deterioration of soil physical structure, although the present data remain correlative rather than directly causal [61]. These findings are also consistent with the observed shift in dominant bud types from tiller and rhizome buds in less degraded meadows to greater relative importance of dicot buds under severe degradation.

#### 3.2.2. Soil Nutrient Availability and Differential Responses of Bud Types

Soil nutrients are pivotal regulators of belowground bud bank dynamics in grassland ecosystems [2,5,62]. Previous studies have shown that fertile soil improves bud diversity by increasing the richness and evenness of bud types, while barren soil reduces bud diversity by asymmetrically enhancing rhizome buds and decreasing tiller buds [9]. Our findings align with these results, where the highest total bud density occurred in moderately degraded meadows, where soil-available nutrients remained relatively high, whereas the lowest bud density occurred in severely degraded meadows, where nutrient levels were strongly depleted. Notably, different bud types did not respond equally to nutrient conditions; available nutrients were positively associated with tiller and rhizome bud densities, whereas their relationships with dicot buds were weak. This result is consistent with experimental studies showing that nitrogen addition can promote rhizome and tiller bud formation in perennial grasses, but has limited or inconsistent effects on dicot buds in forbs [63,64]. A plausible explanation is that bud types differ in their physiological responses to nutrient supply and dormancy regulation [65,66,67]. In contrast, nitrogen addition resulted in a high concentration of ammonium ions in the soil, which exerted detrimental effects on enzyme activity and photosynthetic rate in forbs; consequently, resources allocated to dicot bud formation and sprouting decreased [65].

Our finding that available phosphorus was positively associated with bud bank density differs from some semiarid grassland studies, in which phosphorus addition had little effect on bud bank demography [68]. This discrepancy may reflect differences in nutrient limitations among ecosystems; for example, soil moisture and nitrogen may be the dominant constraints in semiarid grasslands, whereas both nitrogen and phosphorus may limit bud formation and outgrowth in alpine meadows [63,69]. Therefore, the effects of nutrient availability on bud bank dynamics should be interpreted in relation to ecosystem-specific resource limitation, rather than as universally uniform responses.

#### 3.2.3. Soil Microbial Biomass and Differential Responses of Bud Types

In our study, SMBC and SMBN were highest in lightly degraded meadows and declined with increasing degradation intensity, whereas SMBP remained relatively stable from ND to MD meadows but decreased markedly in SD meadows (Figure 5). Furthermore, the SEM results showed positive associations between soil microbial biomass and bud bank density (Figure 7). These patterns suggest that soil microbial biomass may contribute to bud bank persistence, mainly through indirect effects on nutrient turnover and rhizosphere resource availability rather than through direct effects on buds themselves [70,71,72]. Higher microbial biomass is often associated with the enhanced decomposition of soil organic matter and more active carbon and nitrogen transformation, which can increase the supply of resources available for plant growth and bud maintenance [70,71]. In a similar way, microbial phosphorus cycling can alter phosphorus availability in the rhizosphere, and this pathway may help to explain the positive association between SMBP and bud density observed in our study [72]. From this perspective, the positive relationships between microbial biomass and bud density may reflect improved nutrient turnover and more favorable rhizosphere conditions for bud survival and outgrowth [73,74]. In addition, changes in nutrient availability associated with microbial activity may also be related to bud activation through plant physiological regulation, as bud outgrowth is influenced by interactions among nutrient status, auxin, cytokinin, and strigolactone signaling [73,74,75,76]. Previous studies on shoot branching and tiller bud outgrowth further indicate that nitrogen and phosphorus supply can modify physiological pathways related to bud release and elongation [73,74]. Therefore, the role of soil microbial biomass in the present study is best interpreted as an indirect, nutrient-mediated pathway associated with bud bank persistence, rather than as direct causal evidence. However, these mechanisms were not directly measured in the present study and should be interpreted cautiously.

### 3.3. Potential Mechanisms of Soil Property in Regulating Bud Bank Demography Along Meadow Degradation Gradients

Taken together, our results suggest that soil deterioration was closely associated with the decline in bud bank density along the degradation gradient, consistent with previous studies showing that meadow degradation alters soil properties and is accompanied by changes in belowground bud bank dynamics [17,50]. In severely degraded meadows, lower soil water content, higher bulk density, reduced nutrient availability, and lower microbial biomass coincided with markedly reduced bud bank density, supporting earlier evidence that drought, soil compaction, and nutrient limitations constrain bud formation, survival, and outgrowth [5,21,56]. These results indicate that the deterioration of soil physical, chemical, and microbial conditions is an important pathway through which meadow degradation suppresses bud bank persistence [17,19,50,56].

However, soil-mediated effects alone cannot fully explain the observed bud bank dynamics [5,16,59]. Along the degradation gradient, the species contributing to the bud bank changed markedly, and the dominant species in heavily and severely degraded meadows differed substantially from those in non-degraded and lightly degraded meadows [16,59]. In non-degraded and lightly degraded meadows, the bud bank was mainly associated with palatable clonal sedges and grasses, whereas in moderately degraded meadows, several clonal graminoids and forbs contributed substantially to the increase in bud density [16,59]. In contrast, in heavily and severely degraded meadows, the contribution of palatable graminoids declined and the bud bank became increasingly associated with disturbance-tolerant or weedy species, while the overall bud density sharply decreased [5,16,59]. Given that species differ inherently in their clonal growth strategies, bud-bearing organs, bud longevity, dormancy, and intrinsic bud production capacity, these compositional shifts likely contributed substantially to variation in bud bank traits [5]. Therefore, alpine meadow degradation appears to regulate bud bank demography through two linked pathways: one involving deterioration of soil physical, chemical, and microbial conditions that affects bud formation and survival within species, and the other involving grazing-induced species turnover that alters the regenerative traits represented in the community [25,27,59]. Nevertheless, these relationships should still be interpreted with caution; although the RDA and SEM results provide strong evidence for statistical associations among degradation, soil conditions, microbial biomass, bud bank density, and plant functional group biomass, they do not by themselves demonstrate direct causation [19,36,61]. In particular, while the microbial- and nutrient-mediated pathways proposed here are ecologically plausible, they still require direct experimental verification in future studies.

### 3.4. Limitations

Several limitations of this study should be acknowledged. First, some mechanisms discussed here, particularly those involving selective foraging, indirect microbial mediation, and species-specific regeneration strategies, were inferred from previous studies rather than directly tested in the present work. Second, the observed relationships between soil variables, microbial biomass, and bud bank traits were mainly based on field associations and multivariate models, and should thus not be interpreted as direct causal effects. Third, we did not quantify seed-based recruitment, species-level bud turnover, or dormancy dynamics, which may also contribute to variation in bud bank persistence along degradation gradients.

## 4. Materials and Methods

### 4.1. Experimental Site

A long-term ecological experiment was located at Henan Mongol Autonomous County on the eastern edge of the Qinghai–Tibet Plateau (34°44′18″ N, 101°36′31″ E; Qinghai, China), at an elevation of 3523 m. Since 2018, annual field investigations have been carried out in this experimental region to monitor vegetation composition and dynamics. The region features a typical plateau continental monsoon climate, characterized by a mean annual temperature of 0.8 °C and an average annual precipitation of 592 mm.

**Figure 8:**
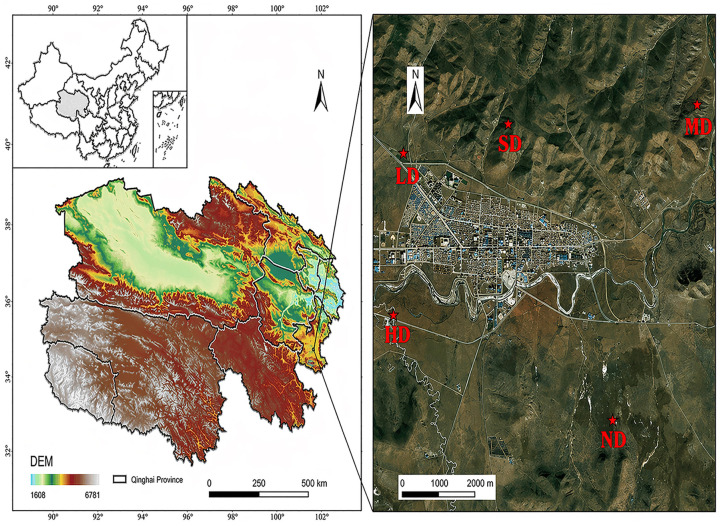
Location of the study area and schematic representation of the spatial arrangement of sampling sites. The left panel indicates the geographical location of the study region in Qinghai Province, China, and its topographic background on the Qinghai–Tibet Plateau. The right panel illustrates the five independent sampling sites used in this study, corresponding to non-degraded (ND), lightly degraded (LD), moderately degraded (MD), heavily degraded (HD), and severely degraded (SD) alpine meadows.

The broader experimental region covered approximately 1000 ha, but the present study did not treat this area as a single sampling site. Instead, five independent sites were selected within the broader experimental region, and one 0.5 ha plot was established at each independent site. Each plot represented one degradation stage, namely, non-degraded meadow (ND), lightly degraded meadow (LD), moderately degraded meadow (MD), heavily degraded meadow (HD), and severely degraded meadow (SD) (Figure 8). The minimum distance between plots was 5 km to enhance spatial independence among sites [77]. The five degradation stages examined in this study were derived from a long-term controlled grazing experiment, rather than from a natural space-for-time chronosequence. At the beginning of the experiment, all plots were fenced and then subjected to different grazing intensities using Tibetan sheep to maintain the target degradation stages. Degradation levels were determined based on vegetation cover and dominant species, following the national standard “Classification Criteria for Degradation, Desertification and Salinization of Natural Grasslands” (GB 19377-2003) [78] and the Qinghai provincial standard “Classification of Alpine Grassland Degradation” (DB63/T 981-2011) [79] (see Table 1). All plots were fenced at the beginning of the experiment to maintain grazing intensities corresponding to different degradation stages and thereby ensure the targeted treatment conditions.

### 4.2. Plant Community Investigation

The vegetation survey was conducted in mid-August 2021. Five random quadrats (0.5 m × 0.5 m) were established in each plot to document species composition and identity, and to measure plant height and coverage [80]. Following the survey, all aboveground biomass, including stems and leaves, was clipped at ground level for each species [81]. To determine dry biomass, the collected samples were dried in an oven at 65 °C for 72 h until constant mass was attained [82].

### 4.3. Bud Bank Investigation

To analyze the composition and abundance of the belowground bud bank in the 0–30 cm soil profile, five soil cores (20 cm × 20 cm × 30 cm) were randomly sampled in situ from alpine meadows representing varying degrees of degradation after investigating the plant community [1]. Maintaining ecological connections between aboveground and belowground subsystems was critical for accurate species identification. Soil cores and the corresponding aboveground vegetation were carefully extracted, transported to the laboratory, and subsequently prepared for analysis. To streamline subsequent experimental procedures, the samples were immersed in a container measuring 25 cm in diameter and 40 cm in height and maintained at room temperature for 24 h. To prevent mechanical injury to the buds, adhering soil was carefully rinsed from the roots using clean water, and entangled root systems were delicately separated with forceps. Species identification was based on morphological characteristics, and the corresponding types and densities of buds associated with each plant species were documented. Based on the classification system established by Qian et al. [2], buds were categorized into three types: rhizome buds were borne on belowground horizontal rhizomes and were connected to elongated internodes that may support lateral spread; tiller buds originated from basal axillary buds at the stem base and usually remained closely attached to the parent shoot with short internodes; and dicot buds arose adventitiously from roots or root collars rather than from stem nodes.

### 4.4. Soil Sampling

From each replicate plot across the degradation gradient, five soil cores were randomly collected with a 3.5 cm diameter corer, sampling the full 20 cm soil profile following vegetation surveys. To ensure the homogeneity of the collected soil samples, 25 individual soils were randomly collected in situ from each degraded gradient plot, and every five were homogenized into a single composite sample, resulting in five replicate homogeneous samples per degradation stage. Soil samples were sieved (2 mm mesh) to remove plant litter, rock fragments, and root debris and then immediately transferred to a cooled container and transported to the laboratory. Each soil sample was divided into two equal parts: one part was air-dried under ambient conditions (dark, ambient temperature) and sieved (1 mm) for nutrient analysis, and the other was frozen at −80 °C for soil microbial biomass carbon, nitrogen, and phosphorus determination [83]. Additionally, within each sampling quadrant, nine soil cores were collected at random locations from the 0–30 cm soil layer at 15 cm intervals for the assessment of soil physical properties using a cylindrical metal sampler with a volume of 100 cm^3^ [83]. To minimize moisture loss, both ends of the cylindrical metal sampler were sealed with plastic caps, and all samplers were promptly placed in a cooling unit and transported to the laboratory [83].

### 4.5. Soil Physical Property Measurement

The uncapped metal cylindrical sampler was immersed in a water-filled flat-bottom container and left submerged for 48 h. The metal cylindrical sampler was weighed after removing the bottom cap, and this mass was recorded as *m*_1_ (g). The uncapped metal cylindrical sampler was then placed in a flat-bottom container filled with dry sand. It was weighed after 2 h and 48 h, and the corresponding masses were recorded as *m*_2_ (g) and *m*_3_ (g), respectively. Finally, the soil sample in the metal cylindrical sampler was oven-dried to a constant weight and weighed to obtain *m*_4_ (g). The soil bulk density (BD, g cm^−3^), soil gravimetric water content (SWC, %), capillary water capacity (CMC, %), field moisture capacity (FMC, %), soil total porosity (STP, %), soil capillary porosity (CP, %), and soil non-capillary porosity (NCP, %) were then calculated as follows [83,84]:

(1) $$\mathrm{BD}\;=\;\frac{m_{4}\;-\;m}{v}$$

(2) $$S\mathrm{WC}\;=\frac{m_{1}\;-\;(m_{4}\;-\;m)}{m_{4\;}-\;m}$$

(3) $$\mathrm{CMC}\;=\frac{m_{2}\;-\;(m_{4}\;-\;m)}{m_{4}\;-\;m}$$

(4) $$\mathrm{FMC}\;=\frac{m_{3}\;-\;(m_{4}\;-\;m)}{m_{4}\;-\;m}\;$$

(5) $$STP\;=\left(1\;-\;\frac{BD}{ds}\right)$$

(6) CP=CMC × BD

(7) NCP=STP − CP

where *v* is the volume of the metal cylindrical sampler (cm^3^), *m* is the empty mass of the metal cylindrical sampler (g), and *ds* is the soil particle density (2.65 g·cm^−3^).

### 4.6. Soil Nutrient Content and Soil Microbial Biomass Measurement

Soil organic carbon (SOC) contents were examined using a total organic carbon analyzer [85]. Soil total nitrogen (TN) and total phosphorus (TP) were digested with H_2_SO_4_ under catalytic conditions using a CuSO_4_:K_2_SO_4_ (1:10) mixture in a digestion furnace at 420 °C for 1 h and 2 h, respectively. Soil TN and TP contents were determined using an Autoanalyzer III continuous flow analyzer (AA3, Bran Luebbe, Norderstedt, Germany). Soil total potassium (TK) content was assessed using flame spectrophotometry [86]. The concentrations of soil-available ammonium nitrogen (NH_4_^+^-N) and nitrate nitrogen (NO_3_^−^-N) were measured after extraction with 2 M KCl solution, followed by colorimetric analysis of the filtrate [87]. Soil microbial biomass carbon (SMBC) and nitrogen (SMBN) were estimated using the chloroform fumigation extraction method, based on the differences in SOC and TN between fumigated and nonfumigated control samples [19]. Soil microbial biomass phosphorus (SMBP) was determined by fumigation with CHCl_3_ and extraction with u NaHCO_3_, followed by estimation using ultraviolet spectrophotometry [88].

### 4.7. Statistical Analysis

A one-way analysis of variance (ANOVA) was employed to compare plant functional group biomass, soil physical properties, nutrient content, and soil microbial biomass between different degraded alpine meadows. The original dataset of bud densities within a 0.5 m × 0.5 m area was converted to 1 m^2^, and the average bud density of each sampling position was subsequently computed and subjected to one-way ANOVA to evaluate variations in bud bank density across degradation levels in the alpine meadows. All data are presented as mean ± standard error.

The relationships between plant functional groups, bud banks, soil physical characteristics, soil nutrients, and soil microbial biomass were examined using a redundancy analysis (RDA) [89]. Furthermore, we developed a structural equation model (SEM) to identify the causal pathways and mediating mechanisms through which soil properties regulate both bud bank structure (including type composition and density) and plant functional biomass at different degradation levels. Prior to conducting the structural equation modeling (SEM), all variables were preliminarily screened through correlation analysis, and those exhibiting high collinearity were removed to avoid multicollinearity issues. The key factors considered in the analysis included biomass of plant functional groups, bud bank density, soil physical properties, nutrient levels, and microbial biomass. Soil physicochemical properties and microbial biomass were designated as initial variables; in contrast, the bud bank type and density and functional group biomass of the plant community were designated as target variables [90]. Statistical analyses were conducted using IBM SPSS Statistics 25.0, with data visualization accomplished through SigmaPlot 14.0. Path modeling was implemented adopting the partial least squares approach (PLS-SEM) in R’s plspm package.

## 5. Conclusions

This study reveals the critical role of belowground bud banks in vegetation regeneration across different levels of degradation. We found that moderate degradation enhances bud bank density, thus promoting regeneration potential, while severe degradation leads to a significant decline in bud bank density despite relatively high aboveground biomass. From a management perspective, our findings indicate that maintaining grazing disturbance within a moderate range may help to sustain both vegetation productivity and belowground regenerative reserves, while severe degradation may reduce long-term recovery potential by depleting persistent bud banks. Therefore, assessments and restorations of degraded alpine meadows should consider belowground bud bank persistence in addition to aboveground vegetation characteristics, especially in communities where relatively high biomass may mask weak regenerative capacity. However, some limitations should be acknowledged. The present study did not quantitatively separate soil-mediated effects from species-composition effects, and some mechanisms discussed here were inferred from previous studies rather than directly tested. In addition, the relative contributions of seed- and bud-based reproduction were not distinguished, and the activity status of different bud types was not separately evaluated. Future studies should address these aspects to better understand vegetation regeneration mechanisms.

## Figures and Tables

**Table 1:** Detailed information for sampling sites in different degraded alpine meadows.

Number of Experimental Sites	Degradation Gradient	Vegetation Cover (%)	Relative Cover of Edible Forages (%)	Dominant Species	Grazing Intensity (Sheep ha^−1^)	Grazing Period	Ecological Interpretation
1	Non-degraded meadow (ND)	>85	>80	*Kobresia capillifolia*, *Elymus nutans*, *Kobresia humilis*	0.00	Ungrazed	Intact alpine meadow dominated by palatable clonal sedges, with high total cover and high relative cover of edible forages.
2	Lightly degraded meadow (LD)	77.5	67.5	*Carex* spp., *Festuca ovina*, *Elymus nutans*	1.33	Year-round	Palatable graminoids still dominate, but both vegetation cover and edible forage cover have begun to decline under light grazing.
3	Moderately degraded meadow (MD)	60	42.5	*Potentilla anserina*, *Elymus nutans*, *Ptilagrostis dichotoma*	4.00	Year-round	Transitional stage characterized by reduced total cover and a marked decline in edible forage cover, with increasing contribution of forbs.
4	Heavily degraded meadow (HD)	40	20	*Potentilla anserina*, *Ligularia virgaurea*, *Poa alpigena*	6.67	Year-round	Community increasingly dominated by unpalatable forbs; both total vegetation cover and edible forage cover are strongly reduced.
5	Severely degraded meadow (SD, black-soil-type)	<30	<10	*Potentilla anserina*, *Morina chinensis*, *Elsholtzia densa*	11.25	Year-round	Typical black-soil-type degraded meadow dominated by weeds/unpalatable forbs, with extremely low total cover and edible forage cover.

## Data Availability

All data are presented in the manuscript.

## Abbreviations

The following abbreviations are used in this manuscript:

BD	Soil Bulk Density
SWC	Soil Gravimetric Water Content
CWC	Capillary Water Capacity
FMC	Field Moisture Capacity
STP	Soil Total Porosity
CP	Soil Capillary Porosity
NCP	Soil Non-Capillary Porosity
SOC	Soil Organic Carbon
TN	Total Nitrogen Contents
TP	Total Phosphorus Contents
TK	Total Potassium Contents
AP	Available Phosphorus
SMBC	Soil Microbial Biomass Carbon
SMBN	Soil Microbial Biomass Nitrogen
SMBP	Soil Microbial Biomass Phosphorus
RDA	Redundancy Analysis
SEM	Structural Equation Model
QTP	Qinghai–Tibet Plateau
ND	Non-Degraded
LD	Lightly Degraded
MD	Moderately Degraded
HD	Heavily Degraded
SD	Severely Degraded
